# Peristeen^**Ⓒ**^ Transanal Irrigation System for Paediatric Faecal Incontinence: A Single Centre Experience

**DOI:** 10.1155/2014/954315

**Published:** 2014-05-06

**Authors:** Omar Nasher, Richard E. Hill, Riyad Peeraully, Ali Wright, Shailinder J. Singh

**Affiliations:** Department of Paediatric Surgery, Queen's Medical Centre, Nottingham University Hospital NHS Trust, Derby Road, Nottingham NG7 2UH, UK

## Abstract

*Aim.* To evaluate the efficacy of the Peristeen^**Ⓒ**^ transanal irrigation system when treating faecal incontinence in children due to chronic idiopathic constipation. *Methods.* A retrospective study was conducted of the first cohort of patients affected with faecal incontinence and referred to our centre for Peristeen^**Ⓒ**^ transanal irrigation treatment between January 2010 and December 2012. Patients with neurogenic bowel disturbance were excluded. A previously described and validated faecal continence scoring system was used to assess bowel function and social problems before and after treatment with Peristeen^**Ⓒ**^. *Results.* 13 patients were referred for Peristeen^**Ⓒ**^ transanal irrigation during the study period. Mean time of using Peristeen^**Ⓒ**^  was 12.6 months (±0.6 months) and mean length of follow-up was 21.2 months (±0.9 months). All patients were noted to have an improvement in their faecal continence score, with a mean improvement from 9.7 ± 1.4 to 14.8 ± 2.7 (*P* = 0.0008) and a reduction in episodes of soiling and increasing in quality of life scores. *Conclusion.* In this initial study, Peristeen^**Ⓒ**^ appears to be a safe and effective bowel management system, which improves bowel function and quality of life in children affected with faecal incontinence as a result of chronic idiopathic constipation, Hirschsprung's disease, and anorectal malformations.

## 1. Introduction

Childhood constipation is a common problem worldwide with an estimated median prevalence in the general population of 8.9% [1]. It can present as difficulty passing stools associated with abdominal pain and infrequent passage of stools and may progress to faecal incontinence due to retention with overflow [2]. In 90% of children, no specific organic cause is found [3] and they are eventually diagnosed with chronic idiopathic constipation (CIC). Faecal incontinence may also occur after definitive surgery for congenital problems such as Hirschsprung's disease (HD) and anorectal malformations (ARM). Chronic constipation can also occur secondary to neuropathic bowel dysfunction due to spina bifida or traumatic spinal cord injuries. CIC has a considerable effect on a patient's psychological and emotional well-being and as such has a substantial impact on patient quality of life [4, 5]. Patients will often have long-term laxative regimes and may require suppositories, behavioural therapy, and sometimes surgical procedures such as manual evacuations, rectal biopsies, and the Malone antegrade continence enema [6]. The Peristeen^*Ⓒ*^ transanal irrigation system involves water irrigation of the large intestine through a disposable balloon catheter and has been successfully employed in the treatment of faecal incontinence in patients with neuropathic bowel dysfunction secondary to spinal cord injuries [7–9]. There is no published literature on its use in treatment of faecal incontinence in children due to CIC, HD, or ARM excluding neuropathic bowel patients. We report the effective use of the Peristeen^*Ⓒ*^ transanal irrigation system for faecal incontinence secondary to CIC, HD, and ARM.

## 2. Methods

A retrospective follow-up study was conducted on the first cohort of patients affected with faecal incontinence and referred to our centre for Peristeen^*Ⓒ*^ transanal irrigation treatment between January 2010 and December 2012. Peristeen^*Ⓒ*^ was offered to all patients who were seeing no improvements on conventional medical therapy, since it became available in our unit in January 2010. Both surgical and nonsurgical management options were explained to parents and patients in depth and those who selected Peristeen^*Ⓒ*^ transanal irrigation treatment were enrolled in the study. These patients would have previously been offered surgery in the form of an antegrade continence enema (ACE) prior to the availability of Peristeen^*Ⓒ*^. The main factor influencing the choice of transanal irrigation was the age and maturity of the patient. In particular, children had to be able to understand and cooperate with the procedure in order to achieve successful results. Patients with constipation due to neuropathic bowel dysfunction (e.g., spinal cord injuries or spina bifida) were excluded from the study. The faecal continence scoring system (Table 1) used previously and validated by Rintala and Lindahl [10] was used to assess bowel function and social issues before and after treatment with Peristeen^*Ⓒ*^. The mean and standard deviation were used to analyse descriptive data and a *t*-test was used to analyse the difference in faecal continence scores before and after Peristeen^*Ⓒ*^.

Patients and their parents were taught how to use the Peristeen^*Ⓒ*^ device during a trial admission onto our surgical ward by our paediatric gastroenterology nurse specialist (AW). We recommended using Peristeen^*Ⓒ*^ as described in the guidelines published by the manufacturing company Coloplast [11]. The families were then supported at home by the local paediatric community nurse who had been educated in the transanal irrigation system. The device was easily accessible to patients, as it is provided by the UK National Health Service on a free of charge prescription. If patients were unhappy with the procedure, they did not have to continue with it. The Peristeen^*Ⓒ*^ system consists of a control unit with a pump, a water bag, and a rectal catheter. Tap water is warmed (36–38°C) and introduced into the colon via the rectal catheter. Once the rectal catheter has been inserted, an inflatable balloon ensures that it remains in situ until the balloon is deflated. The water, along with the stools in the lower portion of the bowel, is then emptied into the toilet [11].

## 3. Results

A total of 13 patients were referred for Peristeen^*Ⓒ*^ during the study period, 3 of which were excluded from the study as they suffered discomfort whilst passing the rectal catheter or simply did not like passing the catheter and decided not to continue with Peristeen^*Ⓒ*^. Two of these had been referred to the psychology service because they refused to take oral medications and the third patient was still continuing on maximal medical therapy, as the parents did not want surgery. The remaining 10 patients (7 males) who underwent Peristeen^*Ⓒ*^ transanal irrigation had underlying diagnoses of CIC in 7, HD in 2, and ARM in 1 (Table 2) and had a mean age of 11.1 ± 2.7 years (age range 10–18 years).

The presenting clinical features were abdominal discomfort, constipation, and faecal soiling in all patients. Previously attempted treatment methods included oral laxatives (both osmotic and stimulant) and per rectum sodium phosphate enemas as per NICE guidelines [6], but these did not result in successful management. In addition, 3 patients required anorectal myectomy, manual evacuation, and intrasphincteric injection of Botulinum toxin.

**Figure 1:**
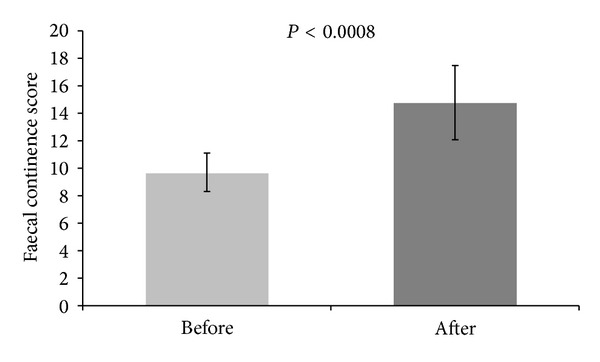
Faecal continence score before and after Peristeen^*Ⓒ*^ treatment.

The mean time of Peristeen^*Ⓒ*^ use was 12.6 months (± 0.6 months) and these patients were followed up for a mean length of 21.2 months (±0.9 months). In each patient, a symptomatic improvement was noted and measured using the faecal continence scoring system [10]. Prior to Peristeen^*Ⓒ*^ treatment, the mean score was 9.7 ± 1.4, whereas after transanal irrigation the mean score was significantly higher at 14.8 ± 2.7 (*P* < 0.0008) (Figure 1). In addition, there was a particular improvement in the frequency of soiling scores and the restrictions in social life scores with 90% having improvement in their “social problems” or quality of life score and 60% achieving a normal score with no social problems following Peristeen^*Ⓒ*^ treatment. No treatment complications were recorded during the follow-up period and none of these patients needed the ACE procedure.

## 4. Discussion

We have demonstrated, albeit in a small study, that Peristeen^*Ⓒ*^ is a safe and effective bowel management system for children with chronic idiopathic constipation not caused by neuropathic bowel disturbance. We have shown a statistically significant improvement in faecal continence score after using the Peristeen^*Ⓒ*^ system and none of the patients who tolerated the transanal irrigation system required a further surgical procedure such as an ACE. A number of studies have looked at Peristeen^*Ⓒ*^ use in patients with neuropathic bowel and demonstrated the benefits of this system [7–9, 12] but we believe this is the first study to exclude neuropathic bowel patients and demonstrate an improvement in faecal continence scores in patients with CIC, HD, and ARMs.

Transanal irrigation has been known since 1500 BC and was initially used in attempts to detoxify the bowel and prevent ileus. In 1987, Shandling and Gilmour demonstrated that faecal continence could be achieved by using enema continence catheters in children with spina bifida affected with faecal incontinence [13]. More recently, the Peristeen^*Ⓒ*^ system has been shown to be effective in the treatment of faecal incontinence in paediatric as well as adult patients with neuropathic bowel dysfunction due to spinal cord injury and spina bifida [7]. In this randomised, controlled, multicenter trial of transanal irrigation versus conservative treatment in spinal-cord-injured patients with neurogenic bowel dysfunction, transanal irrigation improved constipation and faecal incontinence and also ameliorated the quality of life of those patients [7]. In another study of 16 patients with bowel dysfunction secondary to myelomeningocele, transanal irrigation was evaluated by monitoring the intestinal emptying time through radiological imaging. Transanal irrigation was once again proven to be safe and effective and demonstrated to work by increasing intestinal emptying and increasing the progression of the intestinal bolus through the colon [14]. A recent study by Corbett et al. looked at quality of life scores in patients after using the Peristeen^*Ⓒ*^ system and demonstrated that 20 out of 21 had improved quality of life scores [12]. Their study group included patients with myelomeningocele, which our study excluded, and interestingly their median age was 6 years, which is younger than our mean age of 11.1 years. Our mean age is more similar to that reported in other studies demonstrating benefits of Peristeen^*Ⓒ*^ in children with neuropathic bladders: 8.4 years [9] and 12.5 years [8]. We believe that the Peristeen^*Ⓒ*^ is tolerated well by the older patients who are able to understand why they are performing an uncomfortable procedure and tend to be more dedicated and motivated to the task.

There are various qualitative faecal continence scoring systems and quality of life scores, which have been published in the literature. We chose the score developed by Rintala and Lindahl [10], because it had been used and validated in surgical patients. Validated, quantitative scoring systems such as the one used in this study enable a more reliable and accurate statistical analysis [15].

Malone antegrade continence enema procedure is an established surgical treatment option for children with constipation or faecal incontinence [16]. However, it is necessary for the child to undergo a general anaesthetic both to perform the procedure and to close the ACE stoma when it is eventually no longer required. In addition to potential effects on body image, there are well-recognised complications associated with the ACE procedure, with stomal problems including stenosis, leak, and granulation occurring in up to 30% of children [17]. In contrast, no surgical intervention is required for the use of Peristeen^*Ⓒ*^ and the only complication we have witnessed is the potential to cause a mild degree of discomfort. It is documented, however, that irrigation through rectal catheters has a less than 1 in 100,000 chances of causing rectal perforation [18] and we include this information when we have our initial discussions with patients and carers about the Peristeen^*Ⓒ*^ system. It is reasonable to suggest that the response to management using this retrograde irrigation method would also provide evidence of whether the antegrade management of an ACE is likely to be effective; however, as none of our patients who used Peristeen^*Ⓒ*^ needed a further surgical procedure, we cannot confirm this. It is certainly true that, in order for the system to work, it requires dedicated patient and carers.

We believe that this is the first study to assess the response to Peristeen^*Ⓒ*^ transanal irrigation in children with faecal incontinence secondary to CIC, HD, or ARM excluding those with neuropathic bowels. The positive results obtained from this small study are encouraging and point to the need for a larger trial, which we are currently planning.

## Figures and Tables

**Table 1:** Faecal continence scoring system (Rintala and lindahl, 1995, [10]).

Ability to hold back defecation	
Always	3
Problems less than 1/week	2
Weekly problems	1
No voluntary control	0
Feels/reports the urge to defecate	
Always	3
Most of the time	2
Uncertain	1
Absent	0
Frequency of defecation	
Every other day to twice a day	2
More often	1
Less often	1
Soiling	
Never	3
Staining less than 1/week, no change of underwear required	2
Frequent staining, change of underwear often required	1
Daily soiling requires protective aids	0
Accidents	
Never	3
Fewer than 1/week	2
Weekly accidents often require protective aids	1
Daily accidents require protective aids during day and night	0
Constipation	
No constipation	3
Manageable with diet	2
Manageable with laxatives	1
Manageable with enemas	0
Social problems	
No social problems	3
Sometimes (foul odors)	2
Problems causing restrictions in social life	1
Severe social and/or psychic problems	0

**Table 2:** Patient data.

Primary diagnosis	Male	Female
Chronic idiopathic constipation (CIC)	4	3
Anorectal malformations (ARM)	1	
Hirschsprung's disease (HD)	2	

Total	7	3
